# Flexible Diodes/Transistors Based on Tunable p-n-Type Semiconductivity in Graphene/Mn-Co-Ni-O Nanocomposites

**DOI:** 10.34133/2021/9802795

**Published:** 2021-10-13

**Authors:** Lihong Su, Zhou Yang, Xitong Wang, Ziao Zou, Bo Wang, Gary Hodes, Ninghui Chang, Yongyong Suo, Zhibo Ma, Haoxu Wang, Yucheng Liu, Junping Zhang, Shuanhu Wang, Yuefei Li, Fengxia Yang, Jixin Zhu, Fei Gao, Wei Huang, Shengzhong Liu

**Affiliations:** ^1^School of Chemistry and Chemical-Engineering, Northwestern Polytechnical University, Xi'an, 710129 Shaanxi, China; ^2^Dongguan Sanhang Civil-Military Integration Innovation Institute, Dongguan, 52300 Guangdong, China; ^3^Laboratory of Applied Surface and Colloid Chemistry, Ministry of Education; Shaanxi Key Laboratory for Advanced Energy Devices; Shaanxi Engineering Lab for Advanced Energy Technology; Institute for Advanced Energy Materials; School of Materials Science and Engineering, Shaanxi Normal University, Xi'an 710119, China; ^4^School of Aeronautics, Northwestern Polytechnical University, Xi'an, 710072 Shaanxi, China; ^5^Department of Materials and Interfaces, Weizmann Institute of Science, Rehovot 76100, Israel; ^6^Key Lab of Micro/Nano Systems for Aerospace, Ministry of Education, Northwestern Polytechnical University, Xi'an, 710129 Shaanxi, China; ^7^University of Queensland, Australian Institute for Bioengineering & Nanotechnology, Nanomaterials Centre, St. Lucia, Qld, Australia; ^8^School of Physical Science and Technology, Northwestern Polytechnical University, Xi'an, 710129 Shaanxi, China; ^9^Institute of Flexible Electronics, Northwestern Polytechnical University, Xi'an, 710129 Shaanxi, China

## Abstract

We report a novel Mn-Co-Ni-O (MCN) nanocomposite in which the p-type semiconductivity of Mn-Co-Ni-O can be manipulated by addition of graphene. With an increase of graphene content, the semiconductivity of the nanocomposite can be tuned from p-type through electrically neutral to n-type. The very low effective mass of electrons in graphene facilitates electron tunneling into the MCN, neutralizing holes in the MCN nanoparticles. XPS analysis shows that the multivalent manganese ions in the MCN nanoparticles are chemically reduced by the graphene electrons to lower-valent states. Unlike traditional semiconductor devices, electrons are excited from the filled graphite band into the empty band at the Dirac points from where they move freely in the graphene and tunnel into the MCN. The new composite film demonstrates inherent flexibility, high mobility, short carrier lifetime, and high carrier concentration. This work is useful not only in manufacturing flexible transistors, FETs, and thermosensitive and thermoelectric devices with unique properties but also in providing a new method for future development of 2D-based semiconductors.

## 1. Introduction

Modern transistor manufacturing is based on p-n interface phenomena developed in semiconductor science and technology. The migration and separation of electrons/holes in electronic devices are dependent on carrier lifetimes in the devices. Quantum tunneling at the contact interface only involves <2 nm in interface thickness, but the leakage current caused by this tunneling can result in unreliability of the circuit in the manufacture of transistors below 10 nm. Over the past 70 years, metal/semiconductor contacts were divided into two types: Schottky contact and ohmic contact. The formation of a Schottky barrier is based on the mechanism of carrier electron diffusion and thermionic emission, and its properties are determined ideally by the work functions of metal and semiconductor and, in practice, often also by interface defects or reaction [1, 2]. Materials with fixed chemical composition can be p-type, n-type, or intrinsic (or compensated) semiconductors [3, 4]. We found that the work function of negative temperature coefficient (NTC) p-type Mn-Co-Ni-O (MCN) semiconductor nanoparticles is about 0.27 eV higher than that of graphene, which allows electron transfer from graphene to MCN at the contact interface. With the increase of graphene content, the contact between them changes the conductivity of the composite from p-type through electrically neutral to n-type. The contact barrier is dominated by the near-zero electron mass and the volume effect of the nanometer scale MCN particles (Figure S1). There is no leakage of tunneling current base on electronic quantum-tunneling-effect in nanoscale components because of the electron mobile near Dirac point. The development of these new semiconductor composite materials, with inherent flexibility, has broad prospects for the design and manufacture of flexible nanoelectronic components, including design and manufacture of chips, transistors, thermoelectric and Hall components, and sensors [5–20].

## 2. Results

Due to graphene's extremely high electron mobility velocity and zero bandgap characteristics, it is difficult to realize it in semiconducting form without doping or changing the graphene structure. In the past 70 years, contacts between conductors and semiconductors have been divided into ohmic and Schottky contacts. Generally, ohmic contacts have no barrier or only a small one (<a few hundred mV) and are high recombination contacts. The conductivity of MCN at room temperature is about 10^−6^ S/m and that of graphene is about 10^6^ S/m. We measured the MCN work function (WF) and that of graphene by two methods—Kelvin probe (KP) and UPS—and take the average between the two measurements (SI, F). The average WF of graphene is about 4.43 eV while that of the MCN as a ceramic film is about 4.7 eV, (each ±0.05 eV). Therefore, the MCN WF is only ~0.27 eV higher than that of graphene which should result in a close-to-ohmic junction.

**Figure 1:**
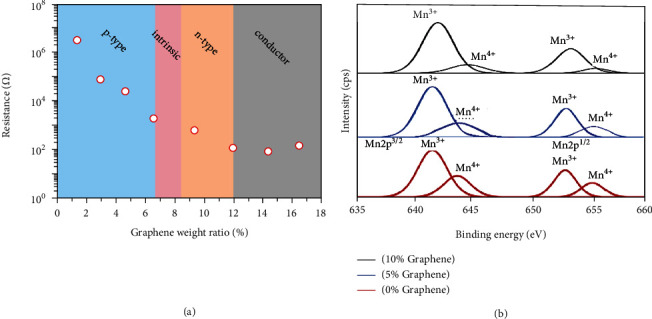
The electrical and chemical properties of complex films with different graphene to MCN ratio. (a) The resistance of 10 *μ*m thick single-layer films at 25°C as a function of different graphene content. (b) Deconvolution of Mn XPS peaks into Mn^3+^ and Mn^4+^ showing how the Mn^3+^ : Mn^4+^ ratio increases with an increase in the graphene content.

We carried out both resistivity and Hall effect measurements (the latter for determining conductivity type and carrier concentration). The contact geometry is shown in Figure S1 of the SI and explained in the corresponding text in the SI; measurements are made both across the films and through it. This directionality may have a strong effect on measured conductivity type and carrier concentrations.  Figure 1(a) shows the resistance values of 10 *μ*m thick single-layer films as a function of graphene content (similar graphs for different film thicknesses are shown in Figure S4). At low graphene concentrations, holes dominate the conductivity. The surface resistance of the composite film decreases nonlinearly (on a semilog plot) when the graphene content increases from zero to about 12% and does not change much when the graphene content increases beyond 12%, when the graphene phase becomes continuous and the film properties are like a conductor. In the intermediate region, the film changes from weak p-type through intrinsic (when a balance is reached between electrons and holes in the film) to increasing n-type as the graphene concentration is increased and finally a conductor dominated by the graphene.

Hall effect measurements show that the MCN-graphene composite films (10 *μ*m thick) change from p-type through intrinsic type to n-type with an increase in the graphene content from 1% to about 13%. The trend of change of resistance and conductivity type of films with different thicknesses is the same, but the critical content of graphene needed to give different conductivity types varies according to thickness. Although the graphene content is less than MCN, the most conductive component in the composite materials is graphene because of its extremely large surface area as well as its much higher conductivity.

The change in Mn^3+^ : Mn^4+^ ratio on addition of graphene to MCN can be seen clearly from XPS measurements (Figure 1(b) and Table S1) where the ratio increases from 2.75 (pure MCN, 0% graphene) to 3.92 (5% graphene) and 5.23 (10% graphene). At ~15% graphene concentration, the Mn signal is no longer clearly visible (Figure S3b), meaning the MCN is essentially completely covered with a multilayer of graphene at that graphene concentration. The data show that the MCN nanoparticle adsorption on the graphene includes both physical and chemical effect.

The (Hall) mobility of the graphene/MCN was measured and found to be 91.4 cm^2^/V·s for 3.5% graphene and 55.0 cm^2^/V·s for 6.5% graphene content (see Table S1, average values). At low graphene concentrations (graphene below 7.5%), the composite shows p-type conductivity while between 7.5% and 12%, it exhibits n-type conductivity (for 10% graphene, the mobility is 0.9 cm^2^/V·s with n-type conductivity). When the graphene content is increased beyond 12.5%, the graphene phase becomes continuous and the properties are like a conductor. The carrier type and concentration of the composite films can be modulated by the graphene MCN ratio and can vary over a wide range. Note that all tests are completed by pressing composite contact particles into thin films at 5-30 MPa with different thicknesses. In addition to the graphene/MCN ratio, the specific conductivity data is also related to the formation pressure and thickness of the thin films. Although there will be fluctuation errors in the test data, the change trend of each sample is the same.

## 3. Discussion

A contact potential difference will occur between any two solid interfaces with different WFs and explains diode rectification. The width of the depletion region determines the thickness of the contact interface. Electron tunneling associated with the contact interface between conductor and semiconductor in general microelectronic devices only involves a distance < 2 nm. The carrier electrons in crystals will be scattered 10^12^-10^13^ times per second, but because of the high electron mobility in graphene, electron scattering is strongly reduced. However, the electron space density of graphene is so large that diffusion is not the main effect [18, 20–24] and the electrons behave like a two-dimensional electronic fluid [25].

**Figure 2:**
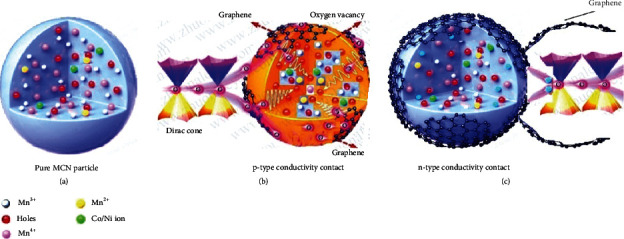
Schematic composition diagram of (a) graphene and MCN nanoparticle composite. (b) Holes in MCN are filled by electrons from graphene. Reducing Mn^4+^ to Mn^3+^ and Mn^2+^ and forming holes in the graphene. (c) With high enough graphene concentration, the graphene's electron dominates the n-type conductivity.

The Schottky contact barrier model is widely used to explain the interface carrier transport mechanism, which is generally based on thermionic emission. Although electron diffusion also occurs, the main contact mechanism between graphene and MCN is dominated by quantum tunneling because of the zero mass electrons of graphene (Figure 2) [22].

The formula of quantum tunneling transmittance is

(1) $$\begin{array}{c} T_{\text{RECTe}}\approx \frac{16E\left(V_{0}-E\right)}{{V_{0}}^{2}}\exp\left(‐\frac{2\text{a}}{\hbar }\sqrt{2m_{\text{e}}\left(V_{0}‐E\right)}\right), \end{array}$$

where the electronic mass = 9.10956 × 10^−31^ kg and the reduced Planck constant *ħ* = 1.05457266 × 10^−34^ j.s. From the work function difference between MCN and graphene, the potential well difference can be assumed to be *V*_0_ − *E* ≈ 0.3 eV approximately. Assuming a potential well *a* = 1 nm, for general electronic calculation, we get

(2) $$\begin{array}{c} T_{\text{RECTe}}\approx 4.8\times 10^{‐19}\times \exp\left(‐4.45\right)\frac{E}{{V_{0}}^{2}}. \end{array}$$

The electron velocity is 1/300 of the speed of light, so the effective dynamic mass, $m=m_{0}/\sqrt{1‐v^{2}/c^{2}}$, *m*_Ge_ = *m* − *m*_0_, *m*_Ge_ is approximately 5.5 × 10^−6^ *m*_0_.

When the potential well width *a* = 1 nm,

(3) $$\begin{array}{c} T_{\text{RECTe}}\approx 4.8\times 10^{‐19}\times \exp\left(‐0.0104\right)\frac{E}{{V_{0}}^{2}}. \end{array}$$

(3)/(2) is 85.6 times. If *a* = 3 nm, 5 nm, and 10 nm, the velocity of graphene electrons entering MCN will drop very rapidly and the effective mass of electrons will increase, but *T*_RECTe_ increases by 310, 334, and 1.0 times at the different values of *a*. Although these assumptions include errors, it can be found that the tunneling transmittance at the contact interface between graphene electrons and MCN can be ~10^2^ times bigger than that of electrons from most other materials (SI tunneling effect calculation part).

However, the real size of MCN nanoparticles is about 10 nm, the actual action width scale of graphene electron tunneling action is above the MCN 10 nm diameter, and the electrons migrate into the whole volume structure of the MCN nanoparticles. For the various Mn valences (4+, 3+, and 2+) in the MCN structure, electrons fill holes resulting in an overall reduction in Mn oxidation state. As discussed above, our XPS data show that the Mn^3+^/Mn^4+^ ratio increases from 2.8 to 5.2 or more as the graphene concentration is increased.

The MCN by itself is p-type as we have already noted. Upon addition of a small amount of graphene (“small” meaning that there is much less than complete coverage of MCN by graphene), the graphene will donate electrons to the high WF MCN (the high electron mobility of monolayer graphene is expected to favor electron transfer to the MCN, particularly where tunneling occurs). This results in two main effects: that of electrons donated to the MCN and holes remaining in the graphene. The effect of electrons on the MCN is to reduce Mn^4+^ to Mn^3+^ (as shown in Figure 1(b)/Table S2). This would be expected to make the MCN even more p-type. However, there are various Ni, Co, and O species that are also present, so it is difficult to predict what happens overall to the MCN, although increasing p-type would be expected in most cases. As the graphene coverage increases and a percolating threshold through graphene forms, conductivity should increase. The conductivity type of the composite in this situation can still be p-type due to the holes in the graphene. As the graphene concentration continues to increase, graphene layers not in direct contact with MCN will conduct by electrons and the conductivity changes to n-type. This is of course very simplified since graphene not in contact with MCN can donate electrons to underlying graphene layers that contained holes; however, the overall picture remains correct. Finally, when the graphene concentration is high enough, the composite will conduct essentially as if it were pure multilayer graphene.

The MCN/graphene composite has many unique characteristics [3, 26]. By pressing composite powders of two different MCN: graphene ratios to form films and stacking the composite films, a flexible p-n junction could be formed due to their different majority carrier types. This was extended to p-n-p or n-p-n transistors by combining three films. Unlike traditional semiconductor devices, electrons are excited from the filled band into the empty band at the graphene Dirac points. These semiconductor devices are based on the two-dimensional electronic fluid characteristics of graphene, where the electrons are very mobile on the two-dimensional surface of the graphene. Potential devices made of this composite thin film include diodes, triodes, and ultraviolet sensors.

**Figure 3:**
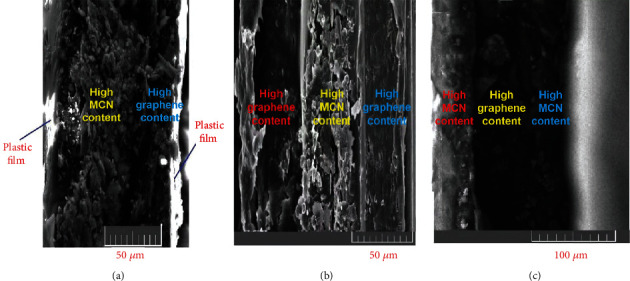
SEM images of cross-sections of films after different multilayer gradient stacking. (a) From left to right, the content of MCN nanometer powder decreases gradually, while the conductivity increases. The darker part of the film has a higher content of graphene and the light parts are the plastic films. (b) From left to right, the content of MCN powder increases gradually; then, on passing the broken green center line, it decreases gradually. (c) From left to right, the content of MCN nanometer powder decreases gradually; then, on passing the broken green line, it increases gradually.

**Figure 4:**
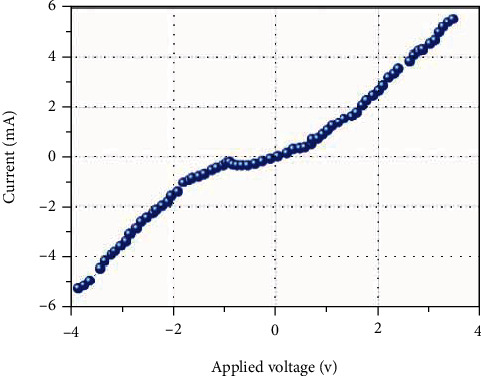
I/V curve of a gradient multilayer thin film p-n junction.

As an example, Figure 3(a) shows the structure and Figure 4 shows an I-V plot of a p-n junction. If two of these p-n junction films are superimposed back-to-back, an n-p-n transistor configuration can be formed (Figure 3(b)) and analogously for a p-n-p configuration (Figure 3(c)) (SI, Figure S5). As a flexible material, the actual gradient films can be assembled into more complex flexible structures and devices of various shapes. This points to a new potential flexible substitute for silicon integrated circuit components.

Because the electron movement in the composite involves tunneling and the graphene's electron transition process is extremely rapid [25, 27–32], transistors manufactured by the composite material can be expected to operate at high frequencies. The carrier mobility of the p-type composite increases with temperature in the range of -50-150°C. This is determined by the properties of MCN itself, and the negative temperature coefficient is proportional to the MCN content. The electron mobility is basically temperature independent in this range because most carriers move on the surface of graphene and the mobility of graphene itself is little affected by temperature. It is found that due to the ultrahigh mobility characteristics of graphene, compared with pure MCN, the composite carrier mobility can be modulated and increased by 10^2^-10^3^ times, and carrier concentration can be adjusted between 10^10^ and 10^19^/cm^3^.

## 4. Conclusions

In conclusion, we have developed a method to effectively regulate p- and n-type semiconductivity of graphene-MCN nanocomposite, making it possible to easily fabricate graphene-based functional semiconductor devices. Unlike traditional semiconductor devices, transistor charge transport is dominated by highly mobile electron movement at the Dirac points on the 2-dimensional surface of graphene. It is anticipated that availability of both p- and n-type graphene-based materials will spur development to fabricate a new generation of semiconductor devices.

## 5. Materials and Methods

**Figure 5:**
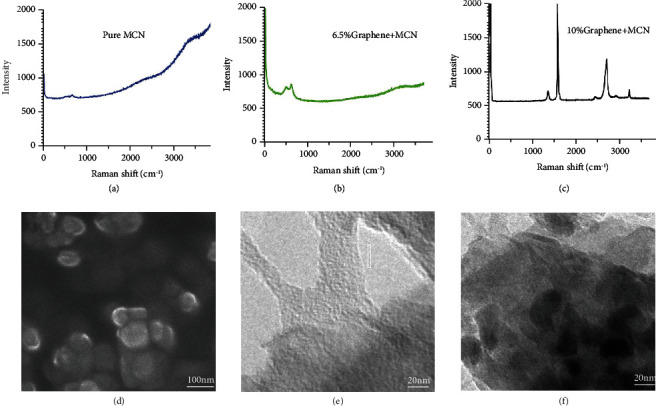
The Raman data, scanning electron microscopy (SEM), and transmission electron microscope images (JSM-7600F instrument and JEM-F200, JEOL Beijing Co., Ltd., Japan). (a) Raman spectrum of pure MCN. (b) Raman spectrum of 6.5% graphene+MCN composite film. (c) Raman spectrum of 10% graphene+MCN composite film. (d) SEM image of MCN nanometer powder. (e) TEM image of graphene. (f) TEM image of MCN nanoparticles coated with graphene (G : MCN mass ratio 1 : 20~25).

MCN nanometer powders (Figures 5(a) and 5(d)) were synthesized by mixing Mn^2+^, Co^2+^, and Ni^2+^ salts with aqueous ammonia and the precipitate fired at 900–1000 K in air for several hours. MCN inorganic semiconductor devices can work stably for a long time with high precision, sensitivity and reliability at high temperatures (up to 780°C), high voltage, and mechanical stress. The transition metal oxide Mn_3-x_Co_2-y_NiO_8-x-y_ (MCN, 0 < *x* + *y* < 0.35) has a spinel structure. The radii of Mn^4+^, Mn^3+^, and Mn^2+^ ions are close to those of Co^3+^, Co^2+^, and Ni^3+^ ions, respectively, which conforms to the Hume-Rothery rule. The MCN nanocrystal structure is compact and stable. MCN is usually a p-type semiconductor with negative temperature coefficient of resistivity in the range of -2%/K to -6%/K. At room temperature, the carrier concentration of these oxides is about 10^9^/cm^3^. It has very low carrier mobility, about 10^−5^ cm^2^/V·s; its intrinsic conductivity is close to that of insulators [18–20], and the negative temperature coefficient depends on the chemical composition of MCN (SI). Co and Ni are used as accepting or doping, and high-temperature sintering forms MCN solid solution with nonstoichiometric defects, which increases the oxygen hole density in the material. Due to Jahn-Teller distortion, some Mn^3+^ ions become Mn^4+^. In the normal temperature equilibrium state, MCN nanocrystals actually have fixed ionic chemical compositions of Mn^4+^/Mn^3+^/Mn^2+^ at room temperature (25°C): Mn^4+^ and Mn^3+^ ions account for more than 80% and are the main components. The size of the individual MCN nanoparticles is about 10 nm (the maximum size of agglomerated particles does not exceed 60 nm, Figure 5(d)), and the specific surface area of the MCN nanoparticles is about 10 m^2^/g. The MCN crystal structure takes Mn_4_Mn_8_O_16_ as the basic unit crystal cell [33, 34]. It can be calculated that particles with the 10 nm MCN account for more than 50% of the interfacial unit crystal cells.

Single-layer graphene (Figure 5(e)) was produced by a micromechanical stripping method (>99% is single-layer graphene, the apparent bulk density is below <0.001 g/cm^3^) [21]. The specific surface area of mechanically exfoliated graphene is about 2630 m^2^/g. Single-layer graphene has an electron energy band structure containing Dirac cones with a zero bandgap. This band structure leads to an effective mass of the two-dimensional surface electrons in graphene that tends to zero, resulting in their very fast velocity of nearly 1/300 of the speed of light. One of its striking properties is the very high mobility ~ up to 150,000 cm^2^/V·s (for single-layer graphene at room temperature) and good electrical conductivity. Current research on semiconducting graphene focuses on heterojunction formation and elemental doping. The direct realization of functional devices by utilizing the Dirac cone characteristics is a topic which is of interest to many researchers [6, 19, 27–32, 35–42].

Graphene and MCN semiconductor powders were weighed according to different mass ratios, (G : MCN between 1 : 5 and 1 : 200 mass ratios). The packing density of graphene, ~0.001 g/ml, is about 1,000 times that of MCN nanopowder (about 1.1 g/ml). MCN powder was added to graphene slowly, and the mixture was ground in a planetary mill for over 36 hours. The average thickness of graphene covering the MCN particles varied from 0.35 to 60 nm depending on the graphene concentration (Figure 4(f)). The composite powder was pressed onto polytetrafluoroethylene (Teflon) film to form a 3 mm square thin film. Raman spectroscopy of these films showed that when the graphene content was less than 8%, the main spectral characteristics of MCN were observed, but the intensity decreased significantly compared with pure MCN (Figures 5(a) and 5(b)). When the graphene content reached 10%, the spectral characteristics of multilayer graphene between MCN nanoparticles were displayed (Figure 5(c)). The composite film is protected by two layers of Teflon membrane, showing excellent bending flexibility (Figure S6). Repeated bending is for more than 500 times, and the fluctuation of performance test data is within 3%.

## Data Availability

All data is available in the main text or the supplementary materials. All materials used in the analysis must be available in some form to any researcher for purposes of reproducing or extending the analysis. Please contact the corresponding author hlshong@nwpu.edu.cn.

## Supplementary Materials

Supplementary Materials Figure S1: XRD pattern of MCN nanometer powder. Figure S2: schematic of the contacts for resistance and Hall measurements. Table S1: the electrical properties of composite films. Figure S3: XPS wide range spectra of (a) pure MCN film. (b) 15% graphene/MCN composite film. Little Mn, Co, or Ni is seen in this spectrum showing high coverage by graphene. Table S2: Mn^3+^/Mn^4+^ contents and ratios for different films. Figure S4: the test curve of MCN negative temperature coefficient. Figure S5: (a, b) typical input and output characteristics of gradient film triode. (c, d) The output characteristic curve of an n-p-n gradient film composite triode (common emitter circuit). IC is the collector current, VOUT is the output voltage, and Ib is the base current. Figure S6: photographs of multilayer gradient film devices under different degrees of bending.
