# Supplementary material for: Flexible Diodes/Transistors Based on Tunable p-n-Type Semiconductivity in Graphene/Mn-Co-Ni-O Nanocomposites
Source: Research (Wash D C). 2021 Oct 13;2021:9802795. doi: 10.34133/2021/9802795 (PMC8532022; doi:10.34133/2021/9802795)
Supplement: Supplementary Materials — Figure S1: XRD pattern of MCN nanometer powder. Figure S2: schematic of the contacts for resistance and Hall measurements. Table S1: the electrical properties of composite films. Figure S3: XPS wide range spectra of (a) pure MCN film. (b) 15% graphene/MCN composite film. Little Mn, Co, or Ni is seen in this spectrum showing high coverage by graphene. Table S2: Mn3+/Mn4+ contents and ratios for different films. Figure S4: the test curve of MCN negative temperature coefficient. Figure S5: (a, b) typical input and output characteristics of gradient film triode. (c, d) The output characteristic curve of an n-p-n gradient film composite triode (common emitter circuit). IC is the collector current, VOUT is the output voltage, and Ib is the base current. Figure S6: photographs of multilayer gradient film devices under different degrees of bending. [file 9802795.f1.zip › Supplementary materials-Re - copy.docx]

**Title**

**Flexible Diodes/Transistors based on Tunable p-n type Semiconductivity in Graphene/Mn-Co-Ni-O Nanocomposites**

**Authors**

Lihong Su^1^^,7*^, Zhou Yang^2^, Xitong Wang^1,7^, Ziao Zou^1^, Bo Wang^1,3^, Gary Hodes^4^, Ninghui Chang^1^, Yongyong Suo^1,3^, Zhibo Ma^1,9^, Haoxu Wang^2,8^, Yucheng Liu^2^, JunpingZhang^1^, Shuanhu Wang^1,4^, Yuefei Li^1,7^, Fengxia Yang^1,7^, Jixin Zhu^5^, Fei Gao^2^, Wei Huang^1,5*^, Shengzhong(Frank) Liu^2*^

**Affiliations**

^1.^School of Chemistry and Chemical-Engineering, Northwestern Polytechnical University, Xi’an, 710129, Shaanxi, China

^2.^Laboratory of Applied Surface and Colloid Chemistry, Ministry of Education; Shaanxi Key Laboratory for Advanced Energy Devices; Shaanxi Engineering Lab for Advanced Energy Technology; Institute for Advanced Energy Materials; School of Materials Science and Engineering, Shaanxi Normal University, Xi’an 710119, China

^3.^School of Aeronautics, Northwestern Polytechnical University, Xi’an, 710072, Shaanxi, China

^4.^ Dept. of Materials and Interfaces, Weizmann Institute of Science, Rehovot 76100, Israel

^5.^ School of Physical Science and Technology, Northwestern Polytechnical University, Xi’an, 710129, Shaanxi, China

^6.^Institute of Flexible electronics, Northwestern Polytechnical University, Xi’an, 710129, Shaanxi, China

^7.^Dongguan Sanhang Civil-Military Integration Innovation Institute, Dongguan, 52300, Guangdong, China

^8.^University of Queensland Univ Queensland, Australian Inst Bioengn&Nanotechnol, NanomatCtr, St Lucia, Qld, Australia

^9.^Key Lab of Micro/Nano Systems for Aerospace, Ministry of Education, Northwestern Polytechnical University, Xi’an, 710129, Shaanxi, China

***Corresponding authors: Prof. Shengzhong Liu( [liusz@snnu.edu.cn](mailto:liusz@snnu.edu.cn)),**

**Prof. Wei Huang [(iamhuang@nwpu.edu.cn)](mailto:(iamhuang@nwpu.edu.cn))**

**Prof. Lihong Su [(hlshong@nwpu.edu.cn)](mailto:(hlshong@nwpu.edu.cn))**

**Supplementary Materials**

1. **Instruments and parameters for experimental processes**
2. (X-ray diffraction analysis, D8 DISCOVER) XRD, Bruker Corporation, U.S.A. CuKα operated at 40kV and 40mA，


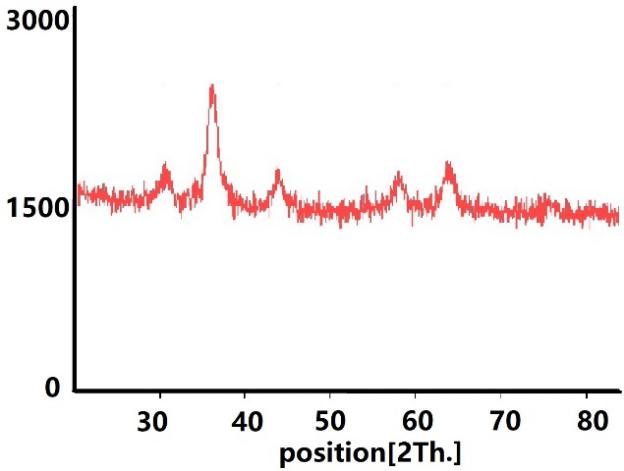


**Fig.S1. XRD pattern of MCN nanometer powder.**

1. Grinding mill (Planetary mill from by ZhongNan milling equipment company, Changsha city, China).
2. Scanning electron microscopy: SEM images were obtained using a JEOLJSM-7600F SEM, JEOL Ltd. Japan, or from an FEI SEM, Helios G4 CX, U.S.A. TEM images from an FEI Titan Krios, U.S.A.
3. Raman spectroscopy Alpha300R，WITec company，Germany.

532 nm TEM00 laser. Laser power > 30 mW, and the Raman wave number is better than 10 cm-1; step size <0.1MW. Spectrometer and detector system：Raman shift range: 10 cm^-1^ ~ 6000 cm^-1^; Spectrometer system: focal length not less than 300 nm; Spectral resolution: better than 1.6cm-1; Spectral repeatability: better than 0.02 cm^-1^.

1. Resistance measurements: Four-point probe (ShenZhen Excellence Instrument & Amp Cooperation, China). American A.S.T.M Standard reference.
2. Work function (WF) measurements: Both Kelvin probe (KP) and UPS were used.

WF was measured with a Bruker Multimode 8 scanning probe microscopy working in KPFM mode. HOPG (WF=4.6 eV) was used as a reference. The measured WF of the MCN particles was 4.70 eV.The WFs of MCN and graphene were also measured by UPS (Thermo Fisher Escalab 250Xi, Analyzer Mode CAE:Pass Energy 2.0eV, energy step size: 0.030eV) using UV photon energy of 21.2 eV. The MCN work function was found to be 4.65 eV, while the graphene was 4.35 eV. Thus, while there is a difference between the values of WF obtained from the two measurements, in both cases, the graphene WF is lower than that of the MCN.

1. X-ray photoelectron spectroscopy, XPS (AXIS Ultra DLD, KRATOS GROUP PLC, Shimadzu company, Japan)
2. Hall effect measurements: Lake Shore 8400 series HMS (Lake Shore Cryotronics, Inc.)

The contact geometry for the resistance (and Hall) measurements is shown in Fig. S2.


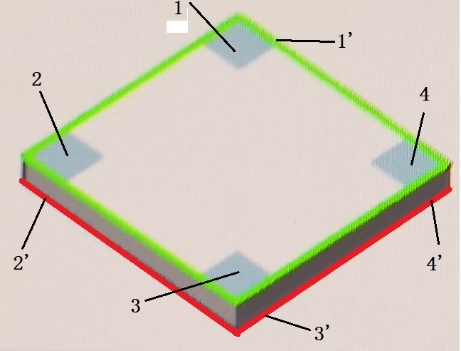


**Fig. S2. Schematic of the contacts for resistance and Hall measurements.**

Points 1, 2, 3, 4 are the upper surface electrode contact points; Points 1', 2', 3', 4' are bottom surface electrode contact points. The electrode is Au or Ag. The connect method is same surface，1-2-3-4 or 1'-2'-3'-4'

**Table.S1 The electrical properties of composite films**

| Graphene content (mass%) | Film Thickness  (µm) | ρ (Ω.m) | Mobility（300K）  (cm^2^/V.s) | type |
| --- | --- | --- | --- | --- |
| 0 | 10 | 5.60x10^7^ | 3x10^-5^ | p |
| 5 | 30 | 2.27x10^7^ | 91.4 | p |
| 6.5 | 30 | 9.68x10^5^ | 55.0 | p |
| 10 | 30 | 2.08x10^3^ | 0.86 | n |

Note: All tests are completed by pressing composite contact particles into thin films with different thicknesses at 5-30 Mpa. In addition to the ratio, the specific conductivity data is also related to the pressing pressure and thickness of the thin films. Although there will be fluctuation error in the test data, the change trend of each sample is the same.

1. Transistor characteristics: Keysight, B1500A semiconductor device analyzer, U.S.A

**2.Comparison of surface area of size 50 nm MCN nanometer particles and same volume of a 1 mm^3^ MCN particle surface area.**

The volume of 1000 µm (assumed sphere) MCN particles is 0.52x10^18^ nm^3^, and that of 50 nm MCN particles is 6.5x10^4^ nm^3^. The volume of 1000 µm MCN particles is equal to that of 8x10^12^ 50 nm particles. The surface area of a 1000 µm diameter particle is 3.14x10^12^ nm^2^, and the total surface area of same volume 50 nm diameter MCN nanoparticles is

50x50x3.14x8x10^12^ =62800x10^12^ nm^2^.

Therefore the total specific surface area increase is 2x10^4^ .

When graphene is coated on the surface of MCN, the effective contact area between them increases greatly compared with graphene coated onto macroscopic MCN.

The diameter of MCN nanoparticles is 10-50 nm. After grinding and mixing with graphene, the distance between the particles is mostly controlled at 1-20 nm. At this scale, tunneling may play a role. Due to the work function difference, holes on the surface and some of the holes inside of the MCN nanoparticles can be filled by the graphene electrons.

**3.****Calculation of Transmission Coefficient of Electron Tunneling Effect when Electrons Contact MCN.**

The electron mass is 9.10956×10^-31^kg kg, 1eV = 1.6× 10^-19^j，reduced Planck constant ħ = 1.05457266x10^-34^j.s The approximate calculation formula of the quantum transmission coefficient(T_recTe_) is as follows:

（1）Assuming a=1 nm, V_0_-E≈0.3 eV, for general electronic calculations:

$T_{RECT\text{e}}\approx\frac{16E(V_{0}-E)}{V_{0}^{2}}\exp\left(\text{-4.45}\right)\text{=4.8}\times10^{\text{-19}}\times\exp\left(\text{-4.45}\right)\frac{E}{V_{0}^{2}}$ （1）

（2）Assuming a=3 nm

$T_{RECT\text{e}}\approx4.8\times10^{\text{-19}}\times\exp\left(\text{-13.35}\right)\frac{E}{V_{0}^{2}}$ (2)

（3）Assuming a=5 nm

$T_{RECT\text{e}}\approx4.8\times10^{\text{-19}}\times\exp\left(\text{-22.25}\right)\frac{E}{V_{0}^{2}}$ (3)

（4）Assuming a=10 nm

$T_{RECT\text{e}}\approx4.8\times10^{\text{-19}}\times\exp\left(\text{-44.51}\right)\frac{E}{V_{0}^{2}}$ (4)

The graphene electron effective mass m_Ge_ is near zero, because the speed of electrons is 1/300 of the speed of light. According to the relativistic mass-velocity relation equation:

$$\text{m}=\frac{m_{0}}{\sqrt{1-\frac{v^{2}}{c^{2}}}}$$

So the effective dynamic mass m_Ge_ =m-m_0_, m_Ge_ is approximately 5.56x10^-6^m_0_.

The transmission coefficient of the graphene electrons can be approximately expressed as:

(5) When a=1 nm

 (5)

(6) Considering that the graphene electron velocity entering MCN will drop rapidly and the effective mass of electrons will increase, assuming that when a=3 nm, m_Ge_ will increase approximately to 0.33333m_0_.

$T_{RECT\text{e}}\approx4.8\times10^{\text{-19}}\times\text{exp（-7.642）}\frac{E}{V_{0}^{2}}$ (6)

(7)Assuming a=5 nm, m_Ge_ increase approximately to 0.556m_0_.

$T_{RECT\text{e}}\approx4.8\times10^{\text{-19}}\text{exp（-16.44）}\frac{E}{V_{0}^{2}}$ （7）

(8)Assuming a=10 nm, m_Ge_ increase approximately to 0.99999m_0_.

$T_{RECT\text{e}}\approx4.8\times10^{\text{-19}}\times\exp\left(\text{-44.49}\right)\frac{E}{V_{0}^{2}}$ （8）

(5)/(1), (6)/(2), (7)/(3),(8)/(4) can be obtained respectively. For different barrier widths a, the transmission coefficient increases by a factor of about 86, 310, 333, 1.02 times respectively. Although these calculation assumptions will contain errors, the calculation results show that the electrons of graphene quantum tunneling effect intensity and range are for larger than those of common materials. The real size of MCN nanoparticles is 10 nm, the interface contact between graphene and MCN nanoparticles actually becomes the three-dimensional volume contact.

**4.****XPS Spectrum of Pure MCN and composite film**


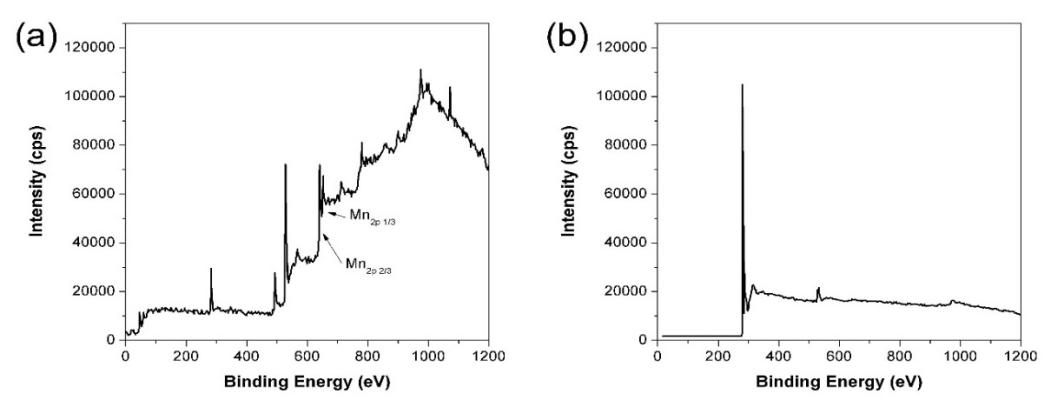


**Fig.S3 XPS wide range spectra of (a) pure MCN film.(b) 15% graphene/MCN composite film. Little Mn, Co or Ni is seen in this spectrum showing high coverage by graphene.**

**Table.S2 Mn^3+^/Mn^4+^ contents and ratios for different films**

**(See Appendix II Excel data)**

|  | **Peak** | **Mass (BE(eV))** | **Mn^3+/^Mn^4+^ mass ratio** |
| --- | --- | --- | --- |
| **Pure MCN** | **Mn 2p-3** | **53.23** | **2.8** |
|  | **Mn 2p-4** | **19.06** |  |
| **5%Graphene+MCN** | **Mn 2p-3** | **55.48** | **3.9** |
|  | **Mn 2p-4** | **14.30** |  |
| **10%Graphene+MCN** | **Mn 2p-3** | **59.74** | **5.2** |
|  | **Mn 2p-4** | **11.60** |  |

Mn^4+^radius is 0.53x10^-10^ m, Mn^3+^ radius is 0.58x10^-10^ m, so the change of ionic valence has little effect on the crystal structure of MCN. The data show the Mn^3+^/Mn^4+^ ratio increases as graphene content increases.

**5.** **Semiconductor characteristics of Pure MCN resistance/temperature relation**





**Fig.S4 The test curve of MCN negative temperature coefficient**

**6.****Transistor property of a MCNG film**

A flexible p-n junction was fabricated by pressing a p-type film onto an n-type one. This was extended to p-n-p or n-p-n transistors by combining three films.

**
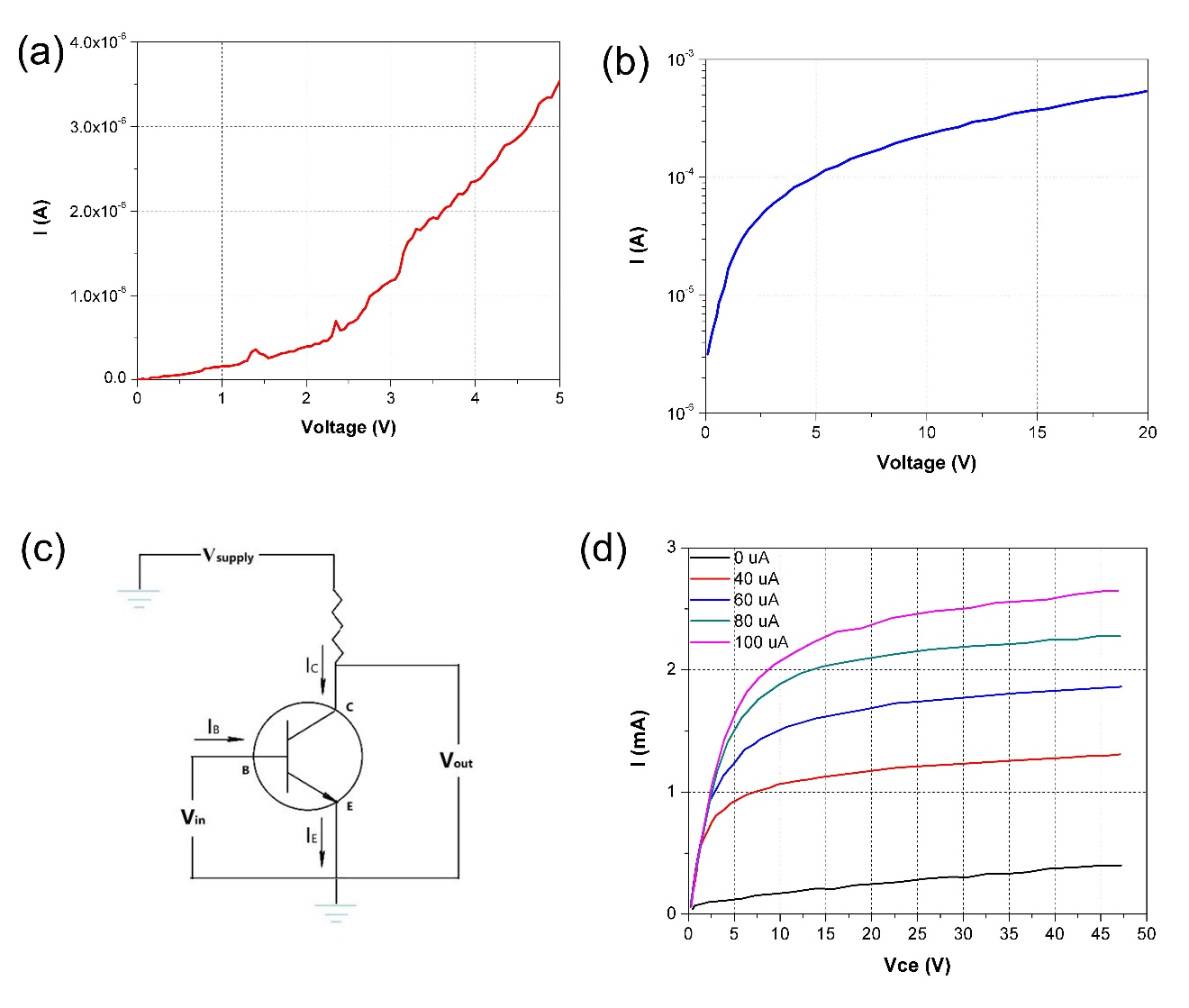
**

**Fig. S5. a, b Typical input and output characteristics of gradient film triode. c, d The output characteristic curve of an n-p-n gradient film composite triode（common emitter circuit,). I_C_ is collector current, V_OUT_ is output voltage and I_b_ is the base current.**

**7.Demonstration of the flexibility of the multiple-layer gradient films**


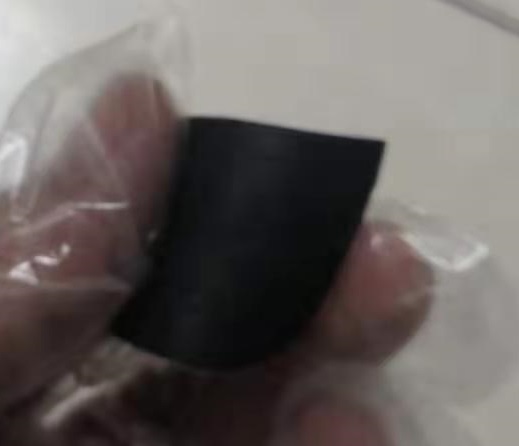

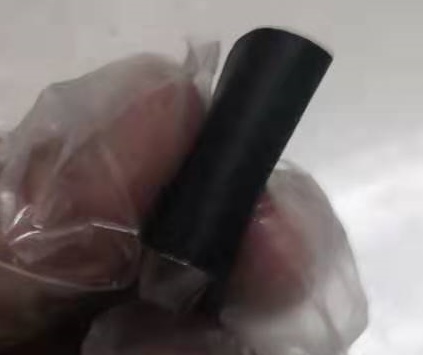


**Fig.S6 Photographs of multilayer gradient film devices under different degrees of bending.**
