# Supplementary figures and images for: Flexible Diodes/Transistors Based on Tunable p-n-Type Semiconductivity in Graphene/Mn-Co-Ni-O Nanocomposites
Source: Research (Wash D C). 2021 Oct 13;2021:9802795. doi: 10.34133/2021/9802795 (PMC8532022; doi:10.34133/2021/9802795)

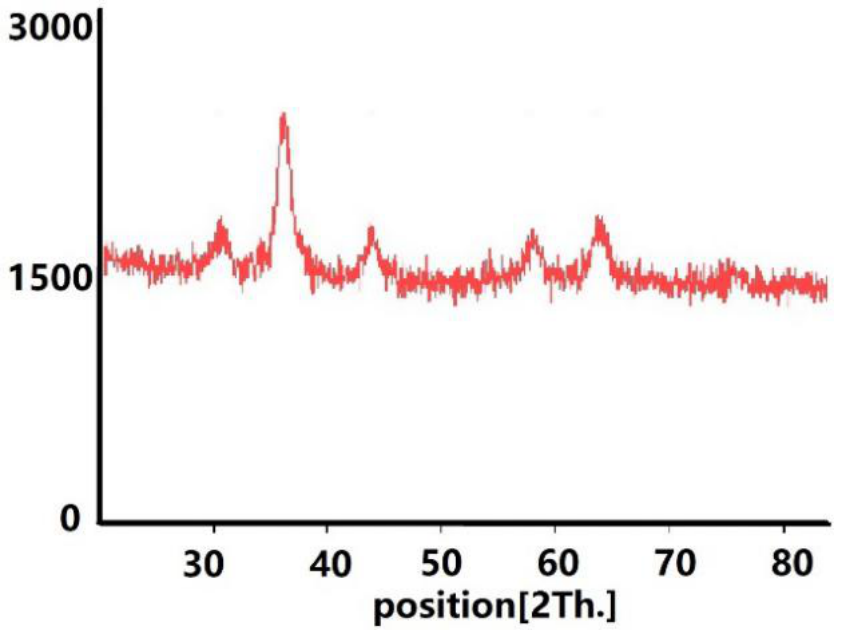

Supplement: Supplementary Materials — Figure S1: XRD pattern of MCN nanometer powder. Figure S2: schematic of the contacts for resistance and Hall measurements. Table S1: the electrical properties of composite films. Figure S3: XPS wide range spectra of (a) pure MCN film. (b) 15% graphene/MCN composite film. Little Mn, Co, or Ni is seen in this spectrum showing high coverage by graphene. Table S2: Mn3+/Mn4+ contents and ratios for different films. Figure S4: the test curve of MCN negative temperature coefficient. Figure S5: (a, b) typical input and output characteristics of gradient film triode. (c, d) The output characteristic curve of an n-p-n gradient film composite triode (common emitter circuit). IC is the collector current, VOUT is the output voltage, and Ib is the base current. Figure S6: photographs of multilayer gradient film devices under different degrees of bending. [file 9802795.f1.zip › Fig.S1.png]

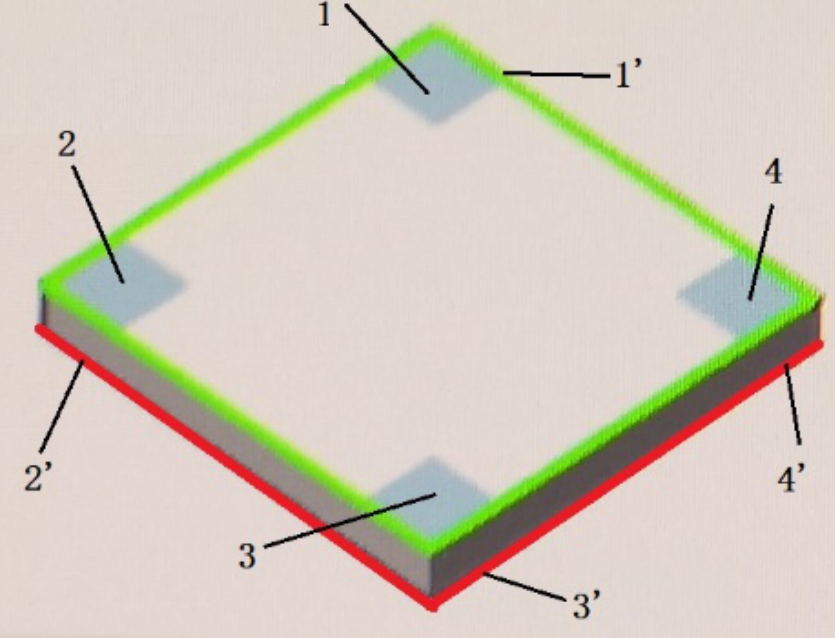

Supplement: Supplementary Materials — Figure S1: XRD pattern of MCN nanometer powder. Figure S2: schematic of the contacts for resistance and Hall measurements. Table S1: the electrical properties of composite films. Figure S3: XPS wide range spectra of (a) pure MCN film. (b) 15% graphene/MCN composite film. Little Mn, Co, or Ni is seen in this spectrum showing high coverage by graphene. Table S2: Mn3+/Mn4+ contents and ratios for different films. Figure S4: the test curve of MCN negative temperature coefficient. Figure S5: (a, b) typical input and output characteristics of gradient film triode. (c, d) The output characteristic curve of an n-p-n gradient film composite triode (common emitter circuit). IC is the collector current, VOUT is the output voltage, and Ib is the base current. Figure S6: photographs of multilayer gradient film devices under different degrees of bending. [file 9802795.f1.zip › Fig.S2.png]

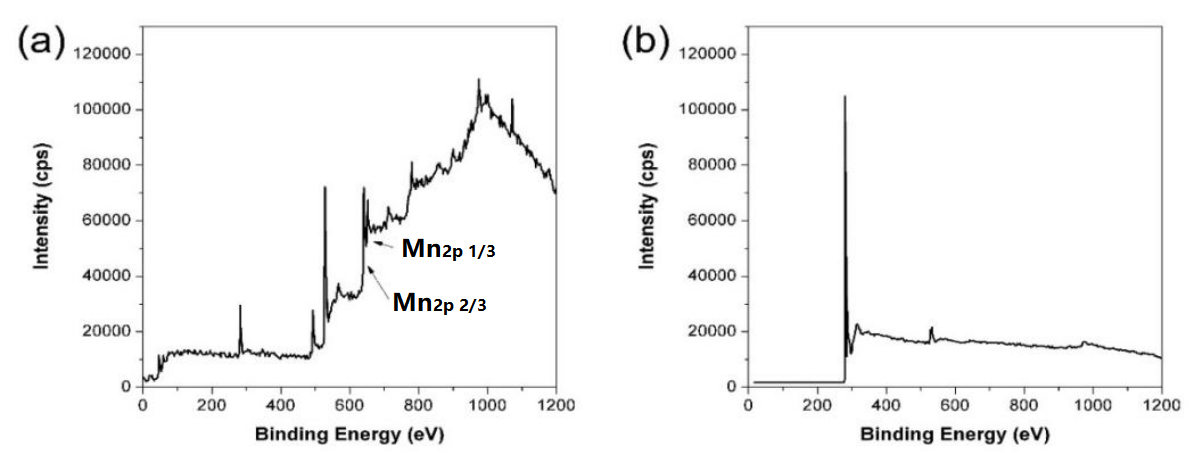

Supplement: Supplementary Materials — Figure S1: XRD pattern of MCN nanometer powder. Figure S2: schematic of the contacts for resistance and Hall measurements. Table S1: the electrical properties of composite films. Figure S3: XPS wide range spectra of (a) pure MCN film. (b) 15% graphene/MCN composite film. Little Mn, Co, or Ni is seen in this spectrum showing high coverage by graphene. Table S2: Mn3+/Mn4+ contents and ratios for different films. Figure S4: the test curve of MCN negative temperature coefficient. Figure S5: (a, b) typical input and output characteristics of gradient film triode. (c, d) The output characteristic curve of an n-p-n gradient film composite triode (common emitter circuit). IC is the collector current, VOUT is the output voltage, and Ib is the base current. Figure S6: photographs of multilayer gradient film devices under different degrees of bending. [file 9802795.f1.zip › Fig.S3.png]

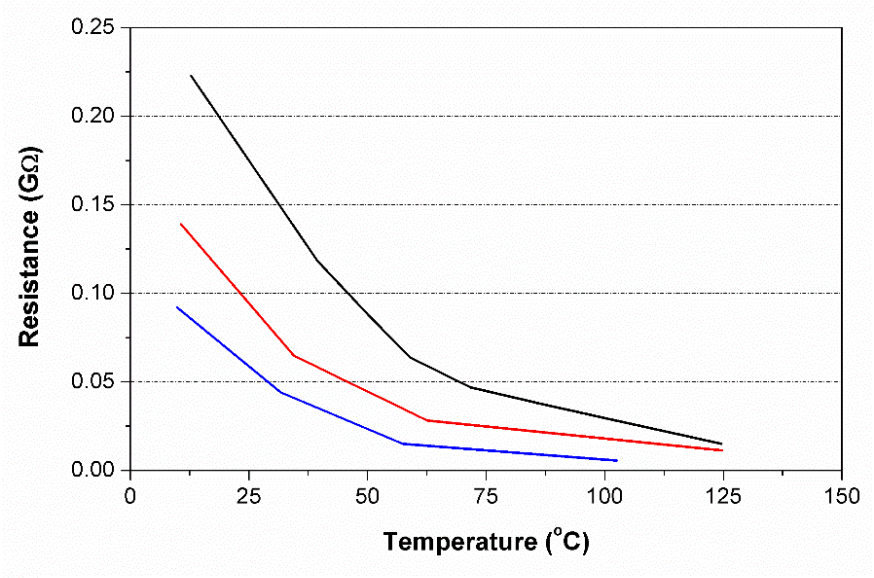

Supplement: Supplementary Materials — Figure S1: XRD pattern of MCN nanometer powder. Figure S2: schematic of the contacts for resistance and Hall measurements. Table S1: the electrical properties of composite films. Figure S3: XPS wide range spectra of (a) pure MCN film. (b) 15% graphene/MCN composite film. Little Mn, Co, or Ni is seen in this spectrum showing high coverage by graphene. Table S2: Mn3+/Mn4+ contents and ratios for different films. Figure S4: the test curve of MCN negative temperature coefficient. Figure S5: (a, b) typical input and output characteristics of gradient film triode. (c, d) The output characteristic curve of an n-p-n gradient film composite triode (common emitter circuit). IC is the collector current, VOUT is the output voltage, and Ib is the base current. Figure S6: photographs of multilayer gradient film devices under different degrees of bending. [file 9802795.f1.zip › Fig.S4.png]

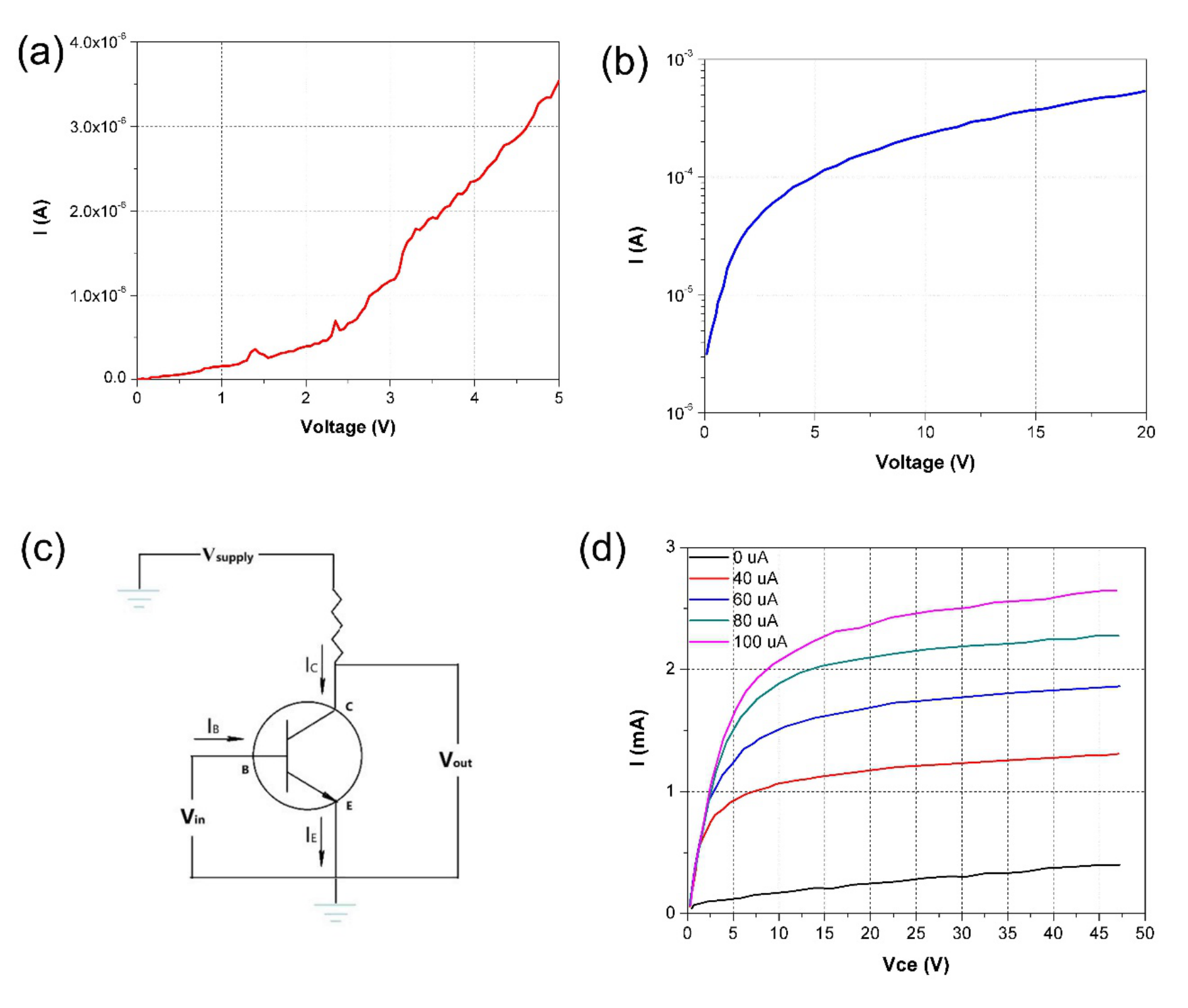

Supplement: Supplementary Materials — Figure S1: XRD pattern of MCN nanometer powder. Figure S2: schematic of the contacts for resistance and Hall measurements. Table S1: the electrical properties of composite films. Figure S3: XPS wide range spectra of (a) pure MCN film. (b) 15% graphene/MCN composite film. Little Mn, Co, or Ni is seen in this spectrum showing high coverage by graphene. Table S2: Mn3+/Mn4+ contents and ratios for different films. Figure S4: the test curve of MCN negative temperature coefficient. Figure S5: (a, b) typical input and output characteristics of gradient film triode. (c, d) The output characteristic curve of an n-p-n gradient film composite triode (common emitter circuit). IC is the collector current, VOUT is the output voltage, and Ib is the base current. Figure S6: photographs of multilayer gradient film devices under different degrees of bending. [file 9802795.f1.zip › Fig.S5.png]

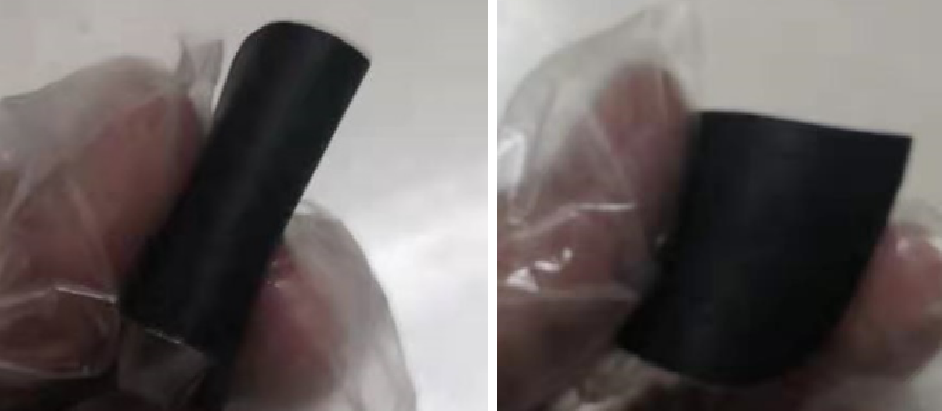

Supplement: Supplementary Materials — Figure S1: XRD pattern of MCN nanometer powder. Figure S2: schematic of the contacts for resistance and Hall measurements. Table S1: the electrical properties of composite films. Figure S3: XPS wide range spectra of (a) pure MCN film. (b) 15% graphene/MCN composite film. Little Mn, Co, or Ni is seen in this spectrum showing high coverage by graphene. Table S2: Mn3+/Mn4+ contents and ratios for different films. Figure S4: the test curve of MCN negative temperature coefficient. Figure S5: (a, b) typical input and output characteristics of gradient film triode. (c, d) The output characteristic curve of an n-p-n gradient film composite triode (common emitter circuit). IC is the collector current, VOUT is the output voltage, and Ib is the base current. Figure S6: photographs of multilayer gradient film devices under different degrees of bending. [file 9802795.f1.zip › Fig.S6.png]
